# Peer Review of “Are We Sure We Fully Understand What an Infodemic Is? A Global Perspective on Infodemiological Problems”

**DOI:** 10.2196/40305

**Published:** 2022-07-21

**Keywords:** communication, conspiracy, COVID-19, education, fake news, infodemic, infodemiology, mass media, public health, risk perception, science

*This is a peer-review report submitted for the paper “Are We Sure We Fully Understand What an Infodemic Is? A Global Perspective on Infodemiological Problems.”*

## Round 1 Review

This paper [1] is a well-written, informative study. However, it has some grammatical errors.
